# Factors Associated with Voluntary Discharge in a Hospital Detoxification Unit: An Observational and Descriptive Analysis

**DOI:** 10.1192/j.eurpsy.2024.311

**Published:** 2024-08-27

**Authors:** P. Gamboa Lozada, G. Ortega Hernández, R. F. Palma Álvarez, A. Ríos Landeo, J. A. Ramos Quiroga, L. Grau López

**Affiliations:** ^1^Department of Psychiatry, Hospital Universitari Vall d’Hebron; ^2^Department of Psychiatry, Hospital Vall d’Hebron, Barcelona, Spain

## Abstract

**Introduction:**

Adherence to treatment for addictive disorders remains a clinical challenge. Despite detoxification admissions being scheduled and initiated voluntarily by the patient, several factors may contribute to treatment discontinuation.^1^ Understanding these factors will enable the development of specific interventions for a more effective approach.^2^

**Objectives:**

To identify and analyze the relationship between specific clinical factors and voluntary treatment discontinuation.

**Methods:**

An observational and descriptive study was conducted using a retrospective database of 1146 patients admitted to the “Hospital Universitari Vall d’Hebron” Detoxification Unit between June 2008 and December 2019. Bivariate analysis was conducted to identify individual associations between clinical factors and voluntary discharge. Subsequently, a multivariate analysis was performed to assess the combined influence of these factors while controlling for potential confounding variables.

**Results:**

A total of 135 patients (11.8%) requested voluntary discharge. Significant differences were found between the voluntary discharge and non-voluntary discharge groups in patients with dual diagnosis (91.1% vs 80.9%, p<0.0001), specifically the presence of psychotic disorder (18.7% vs 12%, p<0.05) and cluster B personality disorder (66.7% vs 31%, p<0.0001). Significant associations were also observed with prior detoxification admissions (64.5% vs 54.1%, p<0.05), heroin as the main admission substance (29.6% vs 13.3%, p<0.0001), lifetime use of more than three substances (65.3% vs 45.3%, p<0.0001), and pre-admission binge-pattern substance use (72.1% vs 51.4%, p<0.0001). A significant relationship was found with therapeutic discharge in the diagnosis of major depressive disorder (14.6% vs. 24.8%, p<0.05), admission for alcohol detoxification (25.9% vs. 42.8%, p<0.0001), and participation in group therapy during admission (27.4% vs. 49.9%, p<0.0001).In the multivariate analysis, it was found that cluster B personality disorder (p<0.0001), heroin as the primary substance of admission (p<0.05), and pre-admission binge-pattern substance use (p<0.05) were independently related to voluntary discharge.

**Conclusions:**

Cluster B personality disorder, admission for heroin detoxification, and pre-admission binge-pattern substance use are factors associated with voluntary treatment discontinuation.

**Disclosure of Interest:**

None Declared

